# Development of a High-Linearity Voltage and Current Probe with a Floating Toroidal Coil: Principle, Demonstration, Design Optimization, and Evaluation

**DOI:** 10.3390/s22155871

**Published:** 2022-08-05

**Authors:** Si-jun Kim, In-ho Seong, Young-seok Lee, Chul-hee Cho, Won-nyoung Jeong, Ye-bin You, Jang-jae Lee, Shin-jae You

**Affiliations:** 1 Applied Physics Lab for PLasma Engineering (APPLE), Department of Physics, Chungnam National University, Daejeon 34134, Korea; 2 Samsung Electronics, Hwaseong-si 18448, Korea; 3 Institute of Quantum Systems (IQS), Chungnam National University, Daejeon 34134, Korea

**Keywords:** plasma diagnostics, plasma monitoring, voltage and current (VI) probe, floating toroidal coil, simulation optimization, VI probe calibration

## Abstract

As the conventional voltage and current (VI) probes widely used in plasma diagnostics have separate voltage and current sensors, crosstalk between the sensors leads to degradation of measurement linearity, which is related to practical accuracy. Here, we propose a VI probe with a floating toroidal coil that plays both roles of a voltage and current sensor and is thus free from crosstalk. The operation principle and optimization conditions of the VI probe are demonstrated and established via three-dimensional electromagnetic wave simulation. Based on the optimization results, the proposed VI probe is fabricated and calibrated for the root-mean-square (RMS) voltage and current with a high-voltage probe and a vector network analyzer. Then, it is evaluated through a comparison with a commercial VI probe, with the results demonstrating that the fabricated VI probe achieved a slightly higher linearity than the commercial probe: $R^{2}$ of 0.9967 and 0.9938 for RMS voltage and current, respectively. The proposed VI probe is believed to be applicable to plasma diagnostics as well as process monitoring with higher accuracy.

## 1. Introduction

Plasma, called the fourth state of matter, consists of physically energetic charged particles (electrons, positive ions, negative ions) and chemically reactive neutral particles (radicals) [1]. Due to their high physical energy and chemical reactivity, plasma has been widely used in various fields such as semiconductor fabrication, medical and environmental industries, aerospace, bio, and nuclear fusion science [2,3]. In particular, in semiconductor fabrication, plasmas significantly influence the plasma etching [4,5,6,7], ashing [8,9], and deposition [10,11] processes to realize feature sizes on the nanoscale. As feature sizes continue to shrink towards a few nanometers with improved levels of integration, process abnormalities such as arcing and leakage that reduce productivity have been regarded as serious problems [12,13,14].

To improve process productivity, process monitoring techniques based on plasma diagnostic methods have garnered much attention [15] since key process parameters such as etching and deposition rates are related to the plasma parameters [16,17,18,19,20]. Plasma diagnostic methods employ an analysis of (i) the current-voltage characteristics of plasma using the Langmuir probe [21,22,23], (ii) the response characteristics of plasma to microwaves using resonators (microwave probes) [24,25,26,27,28,29,30,31,32], (iii) the optical emission characteristics of plasma using an optical emission spectrometer (OES) [33,34], and (iv) the voltage and current (VI) waveforms on a powered electrode using VI probes with circuit modeling [35,36,37].

These diagnostic techniques have been well studied and are commonly used in various research fields. Some of them, however, especially the Langmuir probe and microwave probes, are not suitable for plasma process monitoring, since they are invasive and as a result would distort and be perturbed by the processing. Recently, low-frequency modulation technology and non-invasive types have been proposed and are still under development [27,31,32,38,39]. Commonly implemented plasma process monitoring tools are the OES and VI probe; they are non-invasive and easy to install in the process equipment [40,41,42,43,44]. In general, an OES measures the optical emission from plasma via an optical window and is used for gas composition analysis and anomalous behavior detection. Despite their convenience, however, OESs have limitations in the following three aspects: optical window contamination, narrow spaces of process facilities, and complicated analysis. Process gases such as CF${}_{4}$, C${}_{4}$F${}_{8}$, CHF${}_{3}$, and SiH${}_{4}$ cause optical window contamination that either degrades the emission intensity or cuts off some spectral bands [45], issues for which several techniques have been developed [45,46,47]. Moreover, some process chambers have no optical window since it would perturb process uniformity. Finally, the optical spectra of process gases are highly complicated and pose challenges to analysis since the atomic and molecular spectra overlap, and in certain cases there are no fundamental spectral data for some gases and their compounds [48,49].

The VI probe, in general, measures the voltage and current of the electrode (or antenna) used to generate plasma [35,43,44] and is employed for plasma parameter analysis with some circuit modeling and sensitive detection of anomalous behaviors, especially arcing. As VI probes can be conveniently installed between the electrode (or antenna) and an impedance matcher, they are free from contamination. Nevertheless, since traditional VI probes have separate voltage and current sensors, crosstalk, which is defined as capacitive coupling between the sensors, leads to a degradation of measurement linearity, or in other words, accuracy. To minimize crosstalk, one commonly employed technique is to separate the voltage and current sensors by inserting a metal shield (called a Faraday shield) between them. Lafleur et al. [50] invented a coaxial-type VI probe named the Vigilant probe, where the voltage sensor (called the D-dot antenna) has a conical shape and the current sensor has an axisymmetric groove. Since the current sensor is embedded into external grounded metal and is separated from the voltage sensor, crosstalk can be minimized. In another example, Plasmart Inc. (Daejeon, Korea) [51] developed a printed circuit board (PCB)-type VI probe with a Faraday shield located between the voltage and current sensors to block crosstalk through the inside of the PCB. Despite the Faraday shield, however, crosstalk passing over the PCB still exists. To remove crosstalk completely, Kim et al. [52] developed a VI probe with double walls designed to prohibit the crosstalk passing over as well as through the inside of the PCB. However, in a high power environment, crosstalk can penetrate the Faraday shield, and conventional blocking methods are not effective.

Here, we propose a VI probe with a floating toroidal coil (FTC). Since the FTC plays a role in both voltage and current sensing, the VI probe is free from crosstalk. Through three-dimensional (3D) electromagnetic wave simulation, we first demonstrate the operation principle and establish optimization conditions. Then, based on the optimization results, we fabricated the VI probe and evaluated it with a comparison to a commercial VI probe. The results demonstrate that the fabricated VI sensor has a higher linearity than the commercial probe.

The rest of this paper is organized as follows. The Section 2 provides an explanation and demonstration of the operation principle of the FTC with 3D electromagnetic wave simulation. Design optimization procedures through simulation, and the resultant optimum conditions are also presented. In the Section 3, calibration and evaluation of the fabricated VI probe are investigated. Then, in the Section 4, we summarize the significant results of this paper.

## 2. Principle, Demonstration, and Design Optimization of the VI Probe

### 2.1. Principle of a Floating Toroidal Coil as a Voltage and Current Sensor

**Figure 1:**
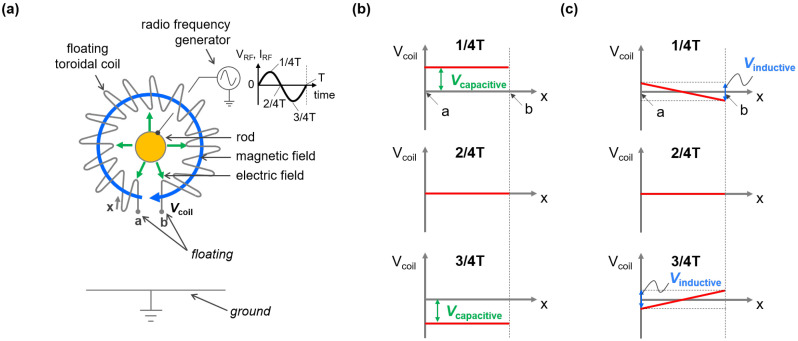
(**a**) Schematic of a floating toroidal coil (FTC). (**b**,**c**) Voltage of the FTC ($V_{\mathrm{coil}}$) when only radio frequency (RF) voltage is applied (**b**) and when only RF current flows through the rod (**c**) at different RF phases (1/4T, 2/4T, and 3/4T), where the T is the period of the RF signal.

In this section, the operation principle of the FTC is qualitatively explored. Figure 1a presents a schematic diagram of the FTC with a cross-sectional view of the signal rod connected to a radio frequency (RF) generator. When RF power is applied to the signal rod, RF voltage is created and RF current flows through the signal rod. For easy understanding, we initially assume two ideal cases: (i) only RF voltage ($V_{\mathrm{RF}}$), and (ii) only RF current ($I_{\mathrm{RF}}$). For the former case, voltage on the FTC is induced by capacitive coupling between the FTC and ground through a time-varying electric field, depicted with green arrows in Figure 1a. Here, capacitive coupling means that the FTC plays a role as a counter-electrode with respect to the rod like a capacitor. Since the RF wavelength is much longer than the dimensions of the FTC, the FTC voltage ($V_{\mathrm{coil}}$) is uniformly distributed between points a and b (Figure 1a) at any RF phase, as shown in Figure 1b; the uniform $V_{\mathrm{coil}}$, therefore, sinusoidally oscillates with time. For the latter RF current-only case, a voltage difference between the FTC ends (a and b, Figure 1a) is induced by inductive coupling between the FTC and the rod through a time-varying magnetic field. Inductive coupling here follows Faraday’s law of induction: an electromotive force is induced to disturb the time-varying magnetic field created by $I_{\mathrm{RF}}$. As shown in Figure 1c, the $V_{\mathrm{coil}}$ is non-uniformly distributed. Note that the center of the FTC acts as a ground and the ends show push/pull characteristics during RF oscillation.

Considering a realistic situation, $V_{\mathrm{RF}}$ and $I_{\mathrm{RF}}$ simultaneously exist. This means that $V_{\mathrm{coil}}$ is induced by a combination of both capacitive and inductive coupling effects. Provided that these effects can be linearly combined (as proved in the next section), the spatiotemporal behavior of $V_{\mathrm{coil}}$ becomes the sum of Figure 1b,c. Therefore, the center $V_{\mathrm{coil}}$ and the different $V_{\mathrm{coil}}$ between the ends represent $V_{\mathrm{capacitive}}$ and $V_{\mathrm{inductive}}$, respectively. Here, $V_{\mathrm{capacitive}}$ and $V_{\mathrm{inductive}}$ mean the magnitude of their couplings, as shown in Figure 1b,c.

Practical use of the FTC to estimate $V_{\mathrm{RF}}$ and $I_{\mathrm{RF}}$ is as follows. We assume that from two points a to b the FTC is symmetric in terms of its center, as shown in Figure 1a. Then, the center $V_{\mathrm{coil}}$ is the same as $V_{\mathrm{capacitive}}$, since $V_{\mathrm{inductive}}$ is zero during RF oscillation at that position (see Figure 1c). Provided that $V_{\mathrm{coil}}$ is symmetrically distributed throughout the FTC, the average value can be the arithmetic mean of the voltages at the ends; hence, $V_{\mathrm{capacitive}}$ is defined as

(1) $$V_{\mathrm{capacitive}}=V_{\mathrm{coil}}^{\mathrm{avg}}=\frac{V_{\mathrm{coil}}^{a}+V_{\mathrm{coil}}^{b}}{2},$$

where $V_{\mathrm{coil}}^{a}$ and $V_{\mathrm{coil}}^{b}$ are the voltages of the FTC at each end (a and b shown in Figure 1a). As $V_{\mathrm{capacitive}}$ results from capacitive coupling, it is noted that the summation of $V_{\mathrm{coil}}^{a}$ and $V_{\mathrm{coil}}^{b}$ can be proportional to $V_{\mathrm{RF}}$ and thus a good indicator to measure $V_{\mathrm{RF}}$ with a coefficient, α, as

(2) $$V_{\mathrm{coil}}^{a}+V_{\mathrm{coil}}^{b}=\alpha V_{\mathrm{RF}}.$$

With a similar perspective, measuring $I_{\mathrm{RF}}$ can be explained as follows. Regarding that the voltage difference of $V_{\mathrm{coil}}$ at the ends originates from inductive coupling, $V_{\mathrm{inductive}}$ is defined as

(3) $$V_{\mathrm{inductive}}=V_{\mathrm{coil}}^{a}-V_{\mathrm{coil}}^{b}.$$

Similar to the above, it is worthwhile to note that here, the subtraction of $V_{\mathrm{coil}}^{b}$ from $V_{\mathrm{coil}}^{a}$ can be proportional to $I_{\mathrm{RF}}$ and thus is a good indicator to measure $I_{\mathrm{RF}}$ with a coefficient, β, as

(4) $$V_{\mathrm{coil}}^{a}-V_{\mathrm{coil}}^{b}=\beta I_{\mathrm{RF}}.$$

Equations (2) and (4) imply that by measuring $V_{\mathrm{coil}}^{a}$ and $V_{\mathrm{coil}}^{b}$, $V_{\mathrm{RF}}$ and $I_{\mathrm{RF}}$ can be assessed, provided that calibration factors α and β are known.

### 2.2. Simulation Demonstration

**Figure 2:**
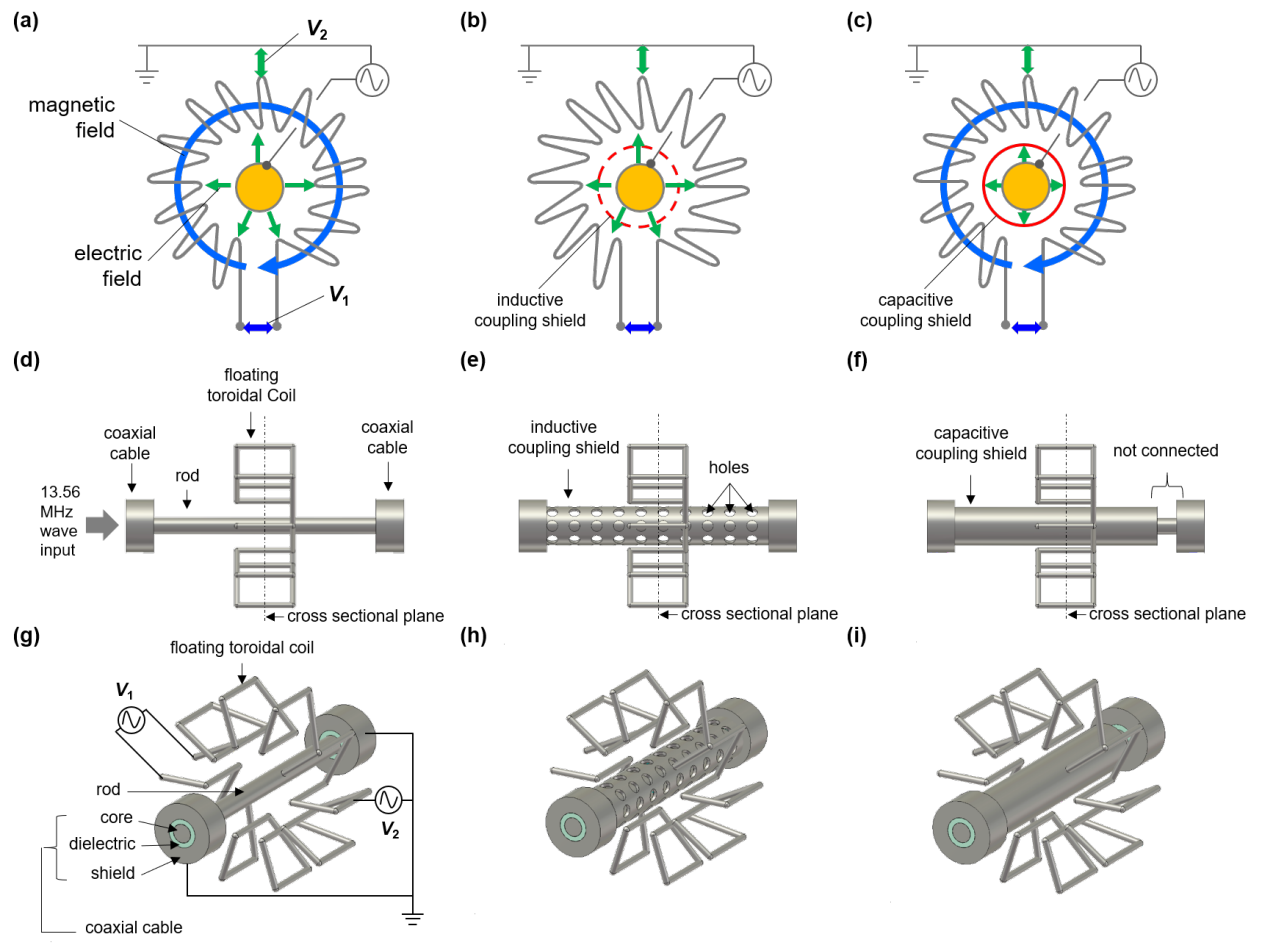
(**a**–**c**) Schematic diagrams of the simulation configurations and (**d**–**i**) corresponding three-dimensional images. Capacitive and inductive coupling is present in (**a**,**d**,**g**); only capacitive coupling is present in (**b**,**e**,**h**); and only inductive coupling is present in (**c**,**f**,**i**). Here, $V_{1}$ means the voltage difference between the ends of the floating toroidal coil, and $V_{2}$ is the voltage difference between the center of the coil and the grounded case.

In this section, we demonstrate the principle introduced in the previous section via 3D electromagnetic wave simulation, CST Microwave Suite [53]. Figure 2a–c show schematic diagrams of three simulation cases: (i) capacitive and inductive coupling (with no shields), (ii) capacitive coupling only (with an inductive coupling shield), and (iii) inductive coupling only (with a capacitive coupling shield). For these three cases, the common configurations are the FTC, the coaxial cables, and the rod, as shown in Figure 2d,g. This apparatus is covered by a rectangular case that is electrically grounded (not depicted in the figure for clarity). The dimensions are listed in Table 1.

The coaxial cables play a role as input and output ports for voltage and current waves. Incident waves from the input port are carried via the rod and induce $V_{\mathrm{coil}}$ on the FTC. In this simulation, a voltage monitor function, which integrates the electric field along a given line, is used to calculate the voltage difference. Here, the voltage monitors $V_{1}$ and $V_{2}$ shown in Figure 2g, respectively, mean the voltage difference between the ends of the FTC, that is $V_{\mathrm{inductive}}$, and between the center of the FTC and the rectangular case, that is $V_{\mathrm{capacitive}}$.

A brief explanation about the role of the inductive coupling shield (ICS) and the capacitive coupling shield (CCS) is as follows. As shown in Figure 2e,h, since the ICS is connected to the coaxial cable shields, which are electrically grounded, a closed current loop from the rod to the ICS forms. Based on Ampere’s law, no net current source exists outside the ICS, since the current in the rod and the shield have the same magnitude but the opposite direction. As a result, no magnetic field outside the ICS can exist, meaning that inductive coupling is blocked. Capacitive coupling in this case exists between the rod and the FTC through the holes in the ICS, as shown in Figure 2e,h. As for the CCS shown in Figure 2f,i, this shield is connected to only one of the coaxial cable shields. In this configuration, no closed current loop can form, meaning that capacitive coupling is blocked while inductive coupling is not.

**Figure 3:**
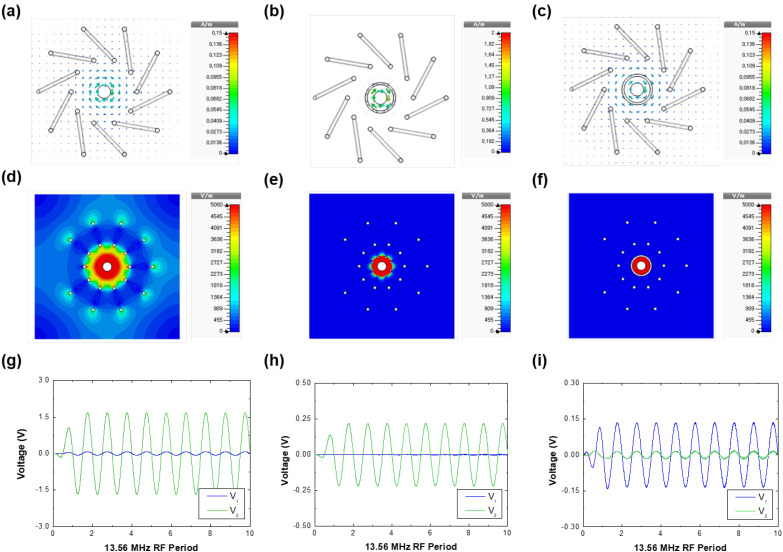
Magnetic field vectors $\vec{B}={\vec{B}}_{x}+{\vec{B}}_{y}+{\vec{B}}_{z}$ (top row), magnitude of electric fields $\left|E\right|=\sqrt{E_{x}^{2}+E_{y}^{2}+E_{z}^{2}}$ (middle row), and voltage waveforms of $V_{1}$ and $V_{2}$ (bottom row) for (**a**,**d**,**g**) capacitive and inductive coupling, (**b**,**e**,**h**) capacitive coupling only, and (**c**,**f**,**i**) inductive coupling only. In the figure, $V_{1}$ means the voltage difference between the ends of the floating toroidal coil, and $V_{2}$ is the voltage difference between the center of the coil and the grounded case.

Simulation results are summarized as follows. Figure 3a–f show the magnetic field vectors and magnitude of the electric field on the cross-sectional plane, respectively, at the phases where their values are maximum. Since magnetic and electric fields form with rotational and diverse directions, respectively, different figure plots (vector and contour) are used for clarity. As for simulation case (i) involving both capacitive and inductive coupling, a rotating magnetic field by RF current in the rod forms inside the FTC, as shown in Figure 3a, demonstrating that the inductive coupling is effective. Furthermore, an electric field strongly forms between the rod and the inner side of the FTC, as shown in Figure 3d, demonstrating that the capacitive coupling is also effective. Since both couplings are effective, the voltage monitors $V_{1}\left(=V_{\mathrm{inductive}}\right)$ and $V_{2}\left(=V_{\mathrm{capacitive}}\right)$ show a sinusoidal waveform signal (Figure 3g). For case (ii) with only capacitive coupling, no magnetic fields are created inside the FTC, since the currents in the rod and in the ICS are opposite (Figure 3b), as explained in the previous paragraph. As shown in Figure 3e, small electric fields escape through the holes (see the green area), which render capacitive coupling effective despite its small magnitude. Furthermore, it is noted that $V_{1}$ is extremely small but $V_{2}$ shows a sinusoidal waveform (Figure 3h), meaning that only capacitive coupling is present. Combining these results, we note that $V_{2}$ can be an indicator of inductive coupling, that is $V_{\mathrm{inductive}}$. As for case (iii) with only inductive coupling, Figure 3c shows that a magnetic field is well produced inside the FTC, similar to Figure 3a, while Figure 3f shows that no electric field forms between the rod and the inner side of the FTC (as electric fields are blocked inside the CCS). This implies that inductive coupling is effective but capacitive coupling is blocked by the CCS. Notably, $V_{1}$ shows a sinusoidal waveform and is much larger than $V_{2}$, as shown in Figure 3i. Hence, $V_{1}$ can be an indicator of $V_{\mathrm{capacitive}}$.

### 2.3. Design Optimization through Simulation

We demonstrated the workings of the FTC in the previous section via simulation. Before fabrication of the proposed sensor for a practical demonstration, it is highly useful to find the optimum conditions to achieve the highest sensitivity also through computer simulation rather than practical trials to minimize development costs. For this, the best method may be to examine all simulation cases for optimization, but this is not recommended due to the simulation cost. Instead, the following procedure is believed to be reasonable [52]. Assuming there are three parameters *a*, *b*, and *c* for optimization, the first step is to sweep the *a* parameter while fixing the other parameters at arbitrary values to find the optimum condition of *a*. The second step sweeps the *b* parameter with the optimized *a* and finds the optimum condition of *b*. The next trial sweeps the *c* parameter with the optimized *a* and *b* and finds the optimum condition of *c*. This process represents one sweeping cycle. By performing several cycles, provided that the optimized conditions of *a*, *b*, and *c* are the same as those of prior sweeping cycles, the final values are the optimum ones.

**Figure 4:**
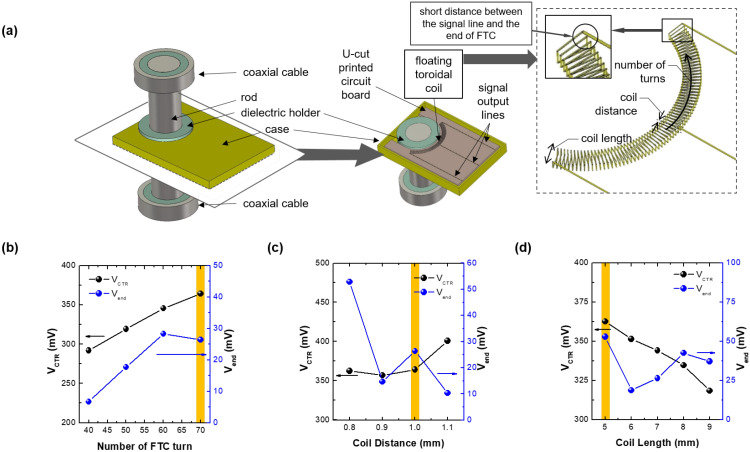
(**a**) Schematic of the VI probe showing the FTC embedded in the PCB, rod, and coaxial lines. The parameters for optimization are illustrated in the dashed box. Magnitude of the voltage difference between the ends of the FTC ($V_{\mathrm{end}}$) and between the center of the coil and ground ($V_{\mathrm{CTR}}$) by number of toroidal coil turns (**b**), coil distance (**c**), and coil length (**d**). The yellow bars highlight the optimum conditions.

Figure 4a shows the simulation configuration of the proposed VI probe and each component: the FTC, U-cut printed circuit board (PCB), signal output lines, rod, dielectric holder, case, and coaxial cables, as well as the parameters for optimization: the number of turns, coil distance, and coil length. The dimensions are listed in Table 2. Here, each signal output line is connected to the two ends and the center of the FTC. The three lines terminate at the end of the U-cut PCB. Three voltage monitors calculate the voltage difference between the case (grounded) and each end of the signal output lines. Based on Equation (1), the center voltage monitor ($V_{\mathrm{CTR}}$) represents $V_{\mathrm{capacitive}}$, and based on Equation (3), the difference between the end voltage monitors ($V_{\mathrm{end}}$s) is $V_{\mathrm{inductive}}$. We introduce the center signal line for an exact measurement of $V_{\mathrm{capacitive}}$. Hence, in this optimization procedure, the optimum condition is defined in terms of the highest signal amplitude of $V_{\mathrm{CTR}}$ and $V_{\mathrm{end}}$ for the fabrication of sensitive VI probe. If their maximum condition is different, the optimum condition is selected with an alternative way: at first, analyzing the tendency of $V_{\mathrm{CTR}}$ and $V_{\mathrm{end}}$ with optimization parameters and then finding the condition where either $V_{\mathrm{CTR}}$ or $V_{\mathrm{end}}$ is the highest value.

Figure 4b shows the amplitude of the $V_{\mathrm{CTR}}$ and $V_{\mathrm{end}}$ waveforms from 40 to 70 turns of the FTC with a coil distance of 1.0 mm and a coil length of 5.0 mm, which are arbitrarily selected. As their maximum conditions are different, the optimum condition is selected with the alternative way. As the number of turns increases, $V_{\mathrm{CTR}}$ monotonically increases since the capacitive coupling area enlarges. On the other hand,$V_{\mathrm{end}}$ is saturated at 60 turns because the effective inductive coupling area inside the FTC becomes saturated. At 70 turns, the signal lines connected to the FTC ends are close to each other, as shown in Figure 4a, while above 70 turns, they are overlapped. Accordingly, the effective number of turns is saturated, and as a result, the optimum condition is 70 turns.

Figure 4c shows the optimization result for the coil distance at the optimized number of turns (70) and a coil length of 5.0 mm. Again, as their maximum conditions are different, the optimum condition is selected with the alternative way. As the coil distance increases, $V_{\mathrm{CTR}}$ gradually increases because the capacitive coupling area is slightly enlarged. Conversely, $V_{\mathrm{end}}$ decreases, except for at a coil distance of 1.0 mm, which results from the decrease in the number of turns per unit length. The opposite trends of $V_{\mathrm{CTR}}$ and $V_{\mathrm{end}}$ imply that the optimum condition is from 0.9 to 1.0 mm. Hence, we choose 1.0 mm as the optimum coil distance since the associated $V_{\mathrm{end}}$ is higher, although the spike of $V_{\mathrm{end}}$ at the 1.0 mm coil distance is not yet well understood.

In the final procedure in one cycle with two optimum conditions (70 turns and 1.0 mm coil distance), as shown in Figure 4d, as the coil length increases, $V_{\mathrm{CTR}}$ decreases while $V_{\mathrm{end}}$ abruptly decreases and then gradually rises. In this case, their maximum conditions are the same, the optimum condition is selected with the highest values of them. Since the outer edges of the FTC get farther away from the rod with increasing coil length, the effective capacitive coupling area decreases, which results in the decrease in $V_{\mathrm{CTR}}$. The abrupt drop of $V_{\mathrm{end}}$ can be explained with the decrease in the number of turns per unit length since the outer arc length increases. The increase in coil length also results in an enlarged area inside the FTC, leading to an enhancement of inductive coupling, which causes the increase in $V_{\mathrm{end}}$. Based on this analysis, while reducing the coil length may seem beneficial, doing so would lead to an overlap of the signal lines at the FTC ends. Hence, the optimum coil length is 5.0 mm.

It is noted that the initial conditions of 1.0 mm coil distance and 5.0 mm coil length at the initial optimization procedure (sweeping the number of turns) are the same as the results from the final optimization procedure. Accordingly, the optimization process is terminated despite the single cycle, and the final conditions are 70 turns, 1.0 mm coil distance, and 5.0 mm coil length. More detailed specifications are listed in Table 3.

## 3. Experiment Results and Discussion

### 3.1. Fabrication

**Figure 5:**
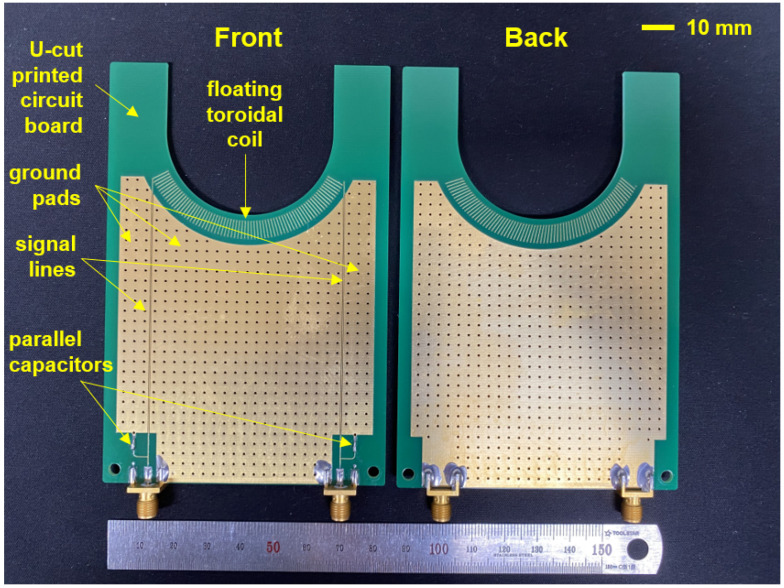
Photograph of the fabricated FTC embedded in a PCB showing both front and back sides.

The fabricated PCB including the FTC, signal lines, and huge ground pads is shown in Figure 5. In the device, we removed the center signal line to minimize the number of signal ports; in fact, $V_{\mathrm{capacitive}}$ can be estimated by measuring the voltages at the FTC ends based on Equation (2). It is important for the VI probe to have high sensitivity, so to minimize RF noise effects, a large grounded pad is attached near the FTC and signal lines. Furthermore, parallel capacitors are installed as a high frequency pass filter, and the signal lines are fabricated as microstrip lines with a characteristic impedance of 50 Ω. Each end of the signal lines is connected with an SMA connector that acts as a signal port.

**Figure 6:**
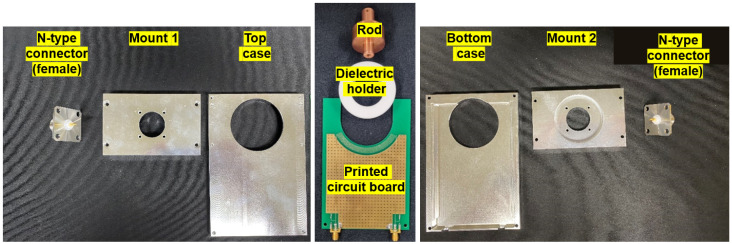
Photographs of the FTC-based VI probe components: N-type connector, mounts, cases, rod, dielectric holder, and PCB.

**Figure 7:**
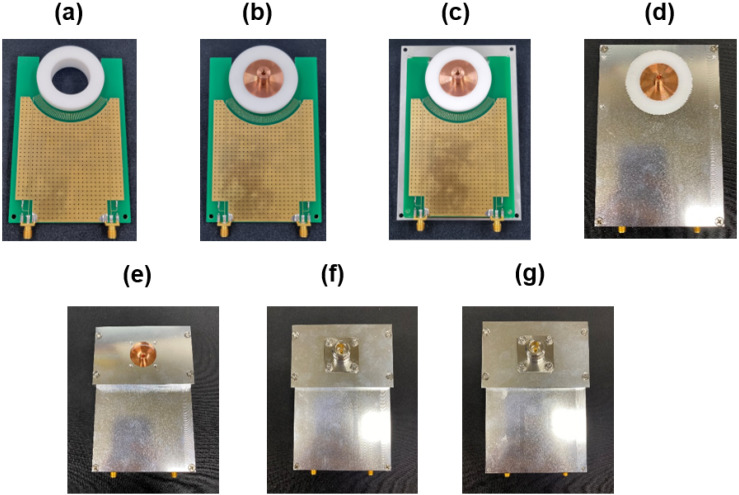
Photographs of the probe assembly procedure. (**a**) The U-cut printed circuit board (PCB) is inserted in the groove of a dielectric holder. (**b**) The rod is placed in the hole inside the dielectric holder. (**c**) The module is mounted on the bottom case. (**d**) The module is covered with the top case. (**e**) Mount 1 is installed. (**f**) An N-type connector is installed. (**g**) Mount 2 and an N-type connector are installed on the back.

Figure 6 shows the components of the fabricated VI probe: N-type connectors, mounts, cases (top and bottom), rod, dielectric holder, and printed circuit board. The N-type connectors coupled with the rod play a role as the input and output ports of the fabricated VI probe. The assembly procedure is described in Figure 7. As shown in Figure 6 and Figure 7, the fabricated VI probe is both easy to assemble and robust.

### 3.2. Calibration

**Figure 8:**
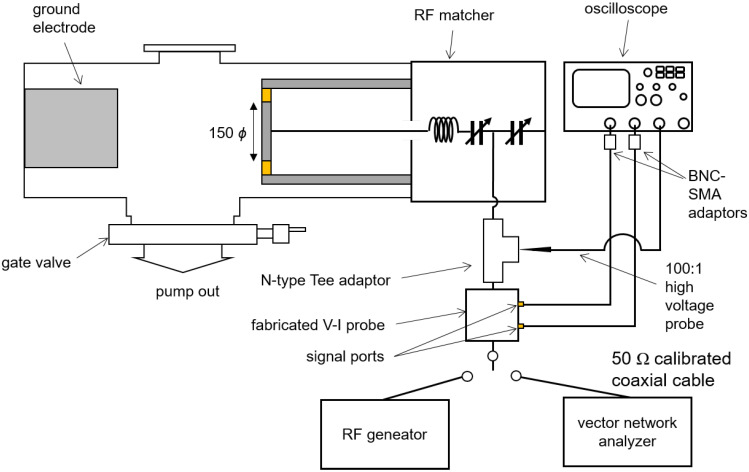
Schematic diagram of the calibration setup for the fabricated FTC-based VI probe. The fabricated VI probe is installed on the input port of the RF matcher with the N-type Tee adaptor. The two signal ports of the fabricated VI probe are connected to channel 1 and 2 of an oscilloscope through coaxial cables with BNC-SMA adaptors.

The experimental setup to identify the coefficients α and β from Equations (2) and (4) is shown in Figure 8. Details of this setup are also described in [26]. For high power calibration, a cylindrical vacuum chamber with a turbomolecular pump (D-35614 Asslar, Pfeiffer Vacuum, Inc., Asslar, Germany) and a rotary pump (GHP-800K, KODIVAC Ltd., Gyeongsan-si, Korea) are employed as the dummy load in this calibration system. The pressure of the vacuum chamber, measured by a vacuum gauge (Baraton, MKS Instruments Inc., Andover, MA, USA), is maintained below 1 mTorr to suppress vacuum discharge causing impedance variation during the calibration procedure; here, the chamber pressure is lower than the minimum measurable range of the vacuum gauge. A cylindrical electrode with a diameter of 150 mm connected with an RF matcher (PathFinder, Plasmart Inc., Daejeon, Korea) is inserted into the vacuum chamber. To minimize impedance variation by thermal effects, coolant flows through the electrode. The fabricated VI probe is installed on the input port of the RF matcher with an N-type Tee adaptor. The two signal ports of the fabricated VI probe are connected to channel 1 and 2 of an oscilloscope (TDS3054B, Tektronix Inc., Beaverton, OR, USA) through coaxial cables with BNC-SMA adaptors. A high-voltage probe (P5100, Tektronix Inc., Beaverton, OR, USA) along with the oscilloscope measures the voltage of the open (left) port of the tee adaptor.

The calibration procedure is as follows. First, we connect a vector network analyzer (E5071B, Agilent Inc., Santa Clara, CA, USA) to the input port of the fabricated VI probe with a coaxial cable with the end calibrated with a kit (SAV20201B, Saluki Technology Inc., Taipei, Taiwan) as shown in Figure 8. Then, the RF matcher is manually manipulated to match the input impedance ($Z_{\mathrm{input}}$) as 50 Ω while the vector network analyzer measures the input impedance. Second, provided that the impedance matching is terminated, the vector network analyzer is replaced with an RF generator (YSR-06MF, Yongshin RF Inc., Hanam-si, Korea). While 13.56 MHz power from 50 W to 300 W is applied to the electrode, the reference voltage ($V_{\mathrm{RF}}$) and current ($I_{\mathrm{RF}}$) are measured by the high-voltage probe and calculated by $I_{\mathrm{RF}}=V_{\mathrm{RF}}/\left|Z_{\mathrm{input}}\right|$, respectively. Each measurement is carried out 20 times.

**Figure 9:**
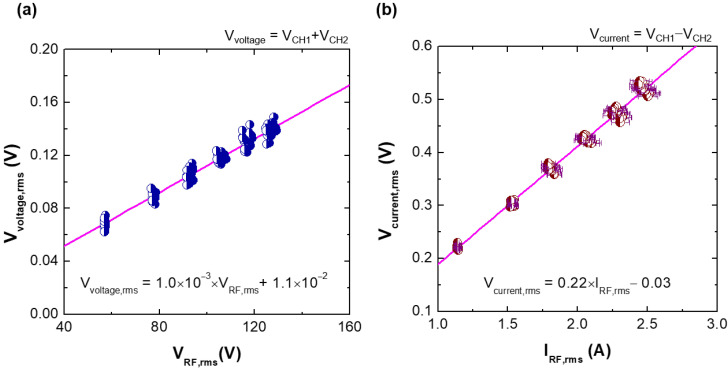
Calibration results of the (**a**) voltage and (**b**) current along increasing RF input voltage and current. To avoid impedance variation by plasma formation during the calibration procedure, the pressure of the vacuum chamber is maintained below 1 mTorr (lower than the minimum measurable range of the vacuum gauge).

Figure 9 shows the root-mean-square (RMS) values of the voltage and current signals from the fabricated VI probe, $V_{\mathrm{voltage},\mathrm{rms}}$ and $V_{\mathrm{current},\mathrm{rms}}$, over the RMS reference voltage and current, $V_{\mathrm{RF},\mathrm{rms}}$ and $I_{\mathrm{RF},\mathrm{rms}}$, respectively. Here, $V_{\mathrm{voltage},\mathrm{probe}}$ is calculated from the RMS value of $(V_{\mathrm{ch}1}+V_{\mathrm{ch}2})/2$, where $V_{\mathrm{ch}1}$ and $V_{\mathrm{ch}2}$ are the voltage waveforms recorded from channel 1 and 2 of the oscilloscope, respectively. Similarly, $V_{\mathrm{current},\mathrm{probe}}$ is from the RMS value of $V_{\mathrm{ch}1}-V_{\mathrm{ch}2}$.

Since RF power is dissipated as heat by each component, such as the electrode, RF matcher, etc., the impedance changes, and this affects the accuracy of calibration. To assess the impedance variance by thermal effects during the calibration procedure, $Z_{\mathrm{input}}$ was measured again after the procedure. The impedance variance is considered to calculate $I_{\mathrm{RF},\mathrm{rms}}$ as the min-max value, represented in Figure 9b as error bars on the x-axis.

### 3.3. Comparison with a Commercial VI Probe

**Figure 10:**
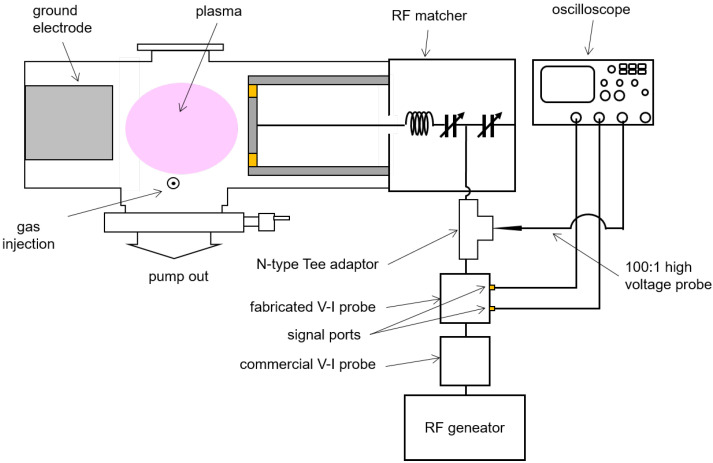
Experimental setup for a comparison of the fabricated VI probe with a commercial VI probe. A commercial VI probe is installed between the RF generator and the fabricated VI probe for the comparison. The RF generator applies power to the electrode and argon plasma is generated.

For an evaluation of the fabricated VI probe via comparison with a commercial VI probe, the experimental setup is slightly changed, as shown in Figure 10. A commercial VI probe (Octive poly, Impedans Ltd., Dublin, Ireland) is installed between the RF generator and the fabricated VI probe for the comparison. A mass flow controller (MFC, TN280, SMTEK Co., Ltd., Seongnam-si, Korea) maintains the flow rate of argon gas at 100 sccm into the vacuum chamber to maintain the chamber pressure at 20 mTorr. The RF generator applies power to the electrode and argon plasma is generated.

**Figure 11:**
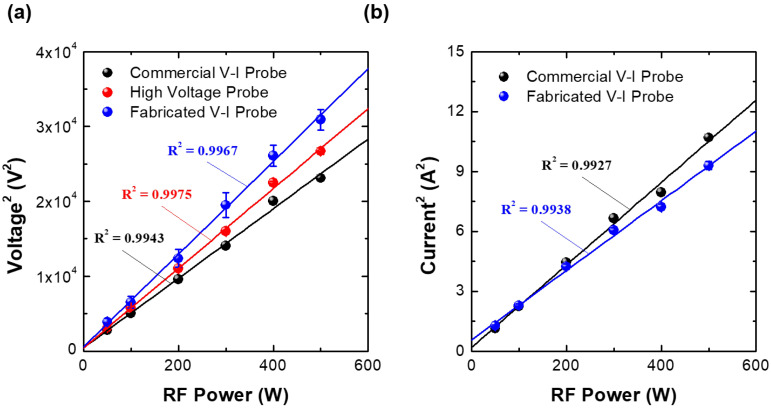
Square of the root-mean-square (RMS) (**a**) voltage and (**b**) current measured by the three probes over RF input power at an argon gas injection rate of 100 sccm, pressure of 20 mTorr, and linearity factors (R-squared values ($R^{2}$)).

Since the RF matcher maintains the source impedance at 50 Ω while the plasma is sustained, the relationships of $V_{\mathrm{RF}}$ and $I_{\mathrm{RF}}$ to the RF power ($P_{\mathrm{RF}}$) are $P_{\mathrm{RF}}=V_{\mathrm{RF}}^{2}/50$ and $P_{\mathrm{RF}}=50I_{\mathrm{RF}}^{2}$, respectively. Figure 11a plots the square of the RMS voltage measured by the fabricated VI probe, the commercial VI probe, and the high-voltage probe with the oscilloscope over input RF power. As the input RF power increases, all probes show a linear increase. Among them, the fabricated VI probe shows a higher $R^{2}$ of 0.9967 for linear fitting than that of the commercial probe. As shown in Figure 11b, the squares of the RMS currents by the fabricated and commercial VI probes also show a linear increase. The fabricated VI probe again shows a higher $R^{2}$ of 0.9938 for the current compared to the commercial probe. In summary, the fabricated VI probe demonstrates a good linearity for both voltage and current, at slightly higher levels than the commercial VI probe.

Here, the squares of the RMS currents from the high-voltage measurement with the oscilloscope is excluded in Figure 11b since it requires the impedance information during plasma discharge. While the RF power is applied, the impedance cannot be measured with the vector network analyzer since the internal impedance of the VNA is 50 Ω and the applied voltage is beyond the measurement limitation of the vector network analyzer.

It should be noted that the voltage level of $V_{\mathrm{voltage},\mathrm{rms}}$ is much lower than $V_{\mathrm{current},\mathrm{rms}}$ based on Figure 9. Traditional VI probes show the opposite characteristic, where the capacitive signal is much larger than the inductive signal as in [52]. This results from the small area of capacitive coupling; traditional voltage sensors use a large area electrode, whereas the FTC consists of wire-type electrodes and naturally has a much smaller coupling area. Further development of the proposed VI probe is therefore important to enhance the capacitive coupling, such as by using other dielectric holders with higher dielectric constants, increasing (decreasing) the radius of the rod (FTC), etc.

The evaluation result for RMS voltage and current does not mean the performance of the proposed probe is better than the commercial probe. The data acquisitions of ten times for each RF power condition in the evaluation process is not enough to exactly compare the proposed VI probe with the commercial probe. Nevertheless, this evaluation result means the successful operation of the prototype. Furthermore, the proposed probe is not fully optimized based on various practical tests; the simulation plays a role in bringing the probe design to near optimized conditions. There are still several practical-test-based optimizations. Later, practical optimization to enhance its performance and exact comparison with the commercial ones will be reported.

## 4. Conclusions

In this paper, we proposed a VI sensor based on a floating toroidal coil. The operation principle of the FTC was demonstrated and its optimum design was established through 3D electromagnetic wave simulation. Here, optimization parameters of the FTC on a printed-circuit board are the number of turns, the coil distance, and the coil length. The resultant optimum conditions are 70 turns, coil distance of 1.0 mm, and coil length of 5.0 mm. Based on the optimum conditions, the proposed VI probe with FTC was fabricated and calibrated based on the high-voltage probe measurement for voltage and the vector network analyzer measurement for the current. During calibration procedure, impedance change by plasma formation and thermal expansion of electrode are suppressed by maintaining pressure of the vacuum chamber below 1 mTorr and flowing coolant through the electrode, respectively. Then, it was evaluated by comparison with a commercial VI probe. The results demonstrated that the FTC-based probe achieved a slightly higher linearity than the commercial one, with an $R^{2}$ of 0.9967 for RMS voltage and 0.9938 for RMS current.

## Figures and Tables

**Table 1:** Dimensions used in the three-dimensional electromagnetic wave simulation. PEC: perfect electric conductor.

Coaxial cable	Outer diameter of core	6 mm
	Conductance of core	infinity (PEC)
	Outer diameter of dielectric	9 mm
	Relative dielectric constant of dielectric	2.1
	Outer diameter of shield	19 mm
	Conductance of shield	infinity (PEC)
	Length	10 mm
Rod	Diameter	6 mm
	Length	80 mm
	Conductance	infinity (PEC)
Floating toroidal coil	Inner diameter	30 mm
	Outer diameter	60 mm
	Width	20 mm
	Wire diameter	2 mm
	Turns	9
	Conductance	infinity (PEC)
Inductive coupling shield (ICS)	Inner diameter	12 mm
	Outer diameter	14 mm
	Hole diameter	4 mm
	Length	80 mm
	Conductance	infinity (PEC)
Capacitive coupling shield (CCS)	Inner diameter	12 mm
	Outer diameter	14 mm
	Length	73 mm
	Conductance	infinity (PEC)
Rectangular case	Volume	100 × 100 × 100 mm${}^{3}$
	Thickness	5 mm
	Conductance	infinity (PEC)

**Table 2:** Dimensions used in the optimization simulation. PEC: perfect electric conductor.

Coaxial cable	Outer diameter of core	30 mm
	Conductance of core	infinity (PEC)
	Outer diameter of dielectric	45 mm
	Relative dielectric constant of dielectric	2.1
	Outer diameter of shield	55 mm
	Conductance of shield	infinity (PEC)
	Length	15 mm
Rod	Diameter	30 mm
	Length	120 mm
	Conductance	infinity (PEC)
Dielectric holder	Inner diameter	30 mm
	Outer diameter	50
	Length	18 mm
	Relative dielectric constant	2.1
Floating toroidal coil	Inner diameter	30 mm
	Outer diameter	60 mm
	Width	20 mm
	Wire diameter	2 mm
	Turns	9
	Conductance	infinity (PEC)
Printed circuit board	Board volume	75.8× 110 × 2.60 mm${}^{3}$
	Pattern thickness	0.07 mm
	Pattern width	0.2 mm
	Pattern conductance	5.96×$0^{7}$ S/m (copper)
Rectangular case	Volume	122 × 86× 15 mm${}^{3}$
	Thickness	2 mm
	Conductance	infinity (PEC)

**Table 3:** Dimensions of the optimized floating toroidal coil.

Optimized floating toroidal coil	Inner diameter	27 mm
	Outer diameter	32 mm
	Coil length	5.0 mm
	Coil distance	1.0 mm
	Turns	70
	Pattern width	0.2 mm
	Pattern height	0.07 mm

## Data Availability

The data presented in this study are available on request from the corresponding author.
